# Supplementary material for: Use of Green Fs Lasers to Generate a Superhydrophobic Behavior in the Surface of Wind Turbine Blades
Source: Polymers (Basel). 2022 Dec 19;14(24):5554. doi: 10.3390/polym14245554 (PMC9781544; doi:10.3390/polym14245554)
Supplement: Supplementary file 1 [file polymers-14-05554-s001.zip › polymers-2058242-supplementary.pdf]

polymers

MDPI

Article

Use of Green Fs Lasers to Generate a Superhydrophobic Behavior in the Surface of Wind Turbine Blades

Joaquín Rivera-Sahún, Luis Porta-Velilla, Germán F. de la Fuente and Luis A. Angurel *

Instituto de Nanociencia y Materiales de Aragón, INMA (CSIC-University of Zaragoza), María de Luna 3, 50018 Zaragoza, Spain

* Correspondence: angurel@unizar.es

Abstract: Ice generation on the surface of wind generator blades can affect the performance of the generator in several aspects. It can deteriorate sensor performance, reduce efficiency, and cause mechanical failures. One of the alternatives to minimize these effects is to include passive solutions based on the modification of the blade surfaces, and in particular to generate superhydrophobic behavior. Ultra-short laser systems enable improved micromachining of polymer surfaces by reducing the heat affected zone (HAZ) and improving the quality of the final surface topography. In this study, a green fs laser is used to micromachine different patterns on the surface of materials with the same structure that can be found in turbine blades. Convenient optimization of surface topography via fs laser micromachining enables the transformation of an initially hydrophilic surface into a superhydrophobic one. Thus, an initial surface finish with a contact angle ca. 69° is transformed via laser treatment into one with contact angle values above 170°. In addition, it is observed that the performance of the surface is maintained or even improved with time. These results open the possibility of using lasers to control turbine blade surface microstructure while avoiding the use of additional chemical coatings. This can be used as a complementary passive treatment to avoid ice formation in these large structures.

Keywords: turbine blades; fs lasers; micromachining; superhydrophobicity

Supplementary Table S1: Examples of different topographies obtained with several laser treatments and the corresponding contact angle measurements.

In the following table, a set of topographies obtained with some selected laser treatments are included, showing also the contact angle measurement.

<table>
<tr><th>TOPOGRAPHIES</th><th>CONTACT ANGLE</th></tr>
<tr><td colspan="2">E_{pulse} = 48.5 µJ/pulse, v_{laser} = 150 mm/s, $\langle F_{1D} \rangle$ = 6.5 J/cm², d_{lines} = 200 µm</td></tr>
<tr><td>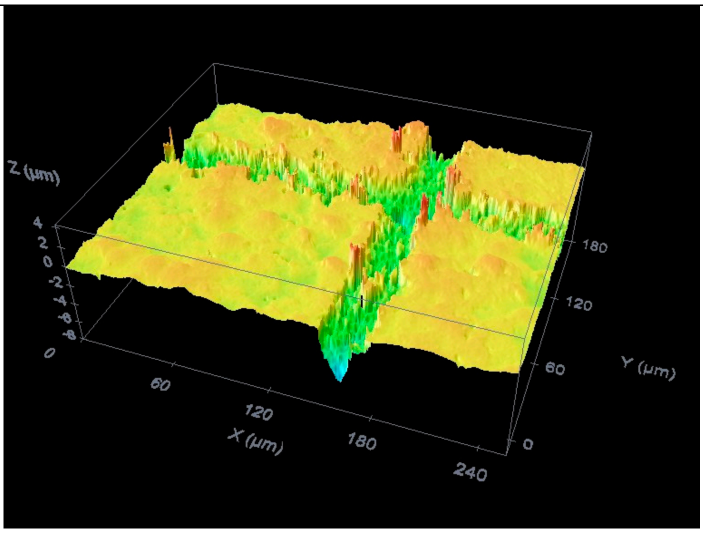
</td><td>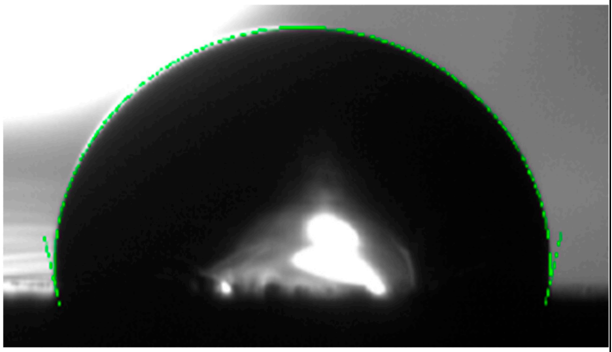</td></tr>
<tr><td colspan="2">E_{pulse} = 48.5 µJ/pulse, v_{laser} = 150 mm/s, $\langle F_{1D} \rangle$ = 6.5 J/cm², d_{lines} = 35 µm</td></tr>
<tr><td>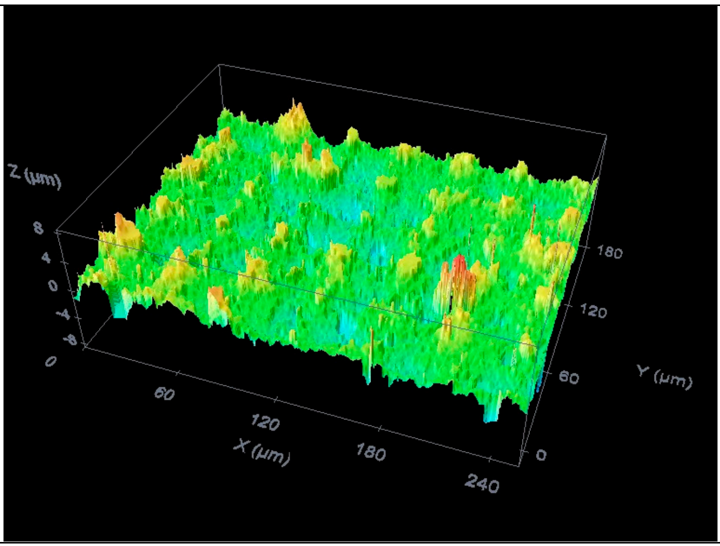
</td><td>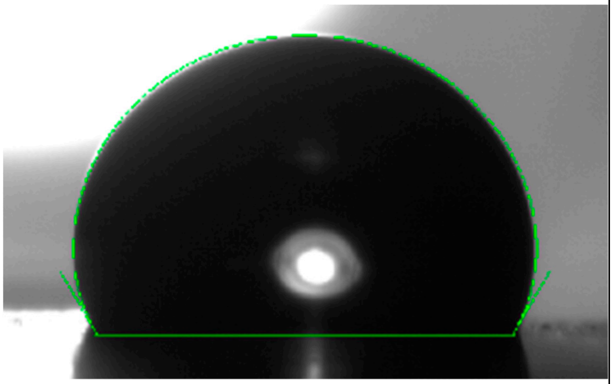</td></tr>
<tr><td colspan="2">E_{pulse} = 48.5 µJ/pulse, v_{laser} = 9 mm/s, $\langle F_{1D} \rangle$ = 107,7 J/cm², d_{lines} = 60 µm</td></tr>
<tr><td>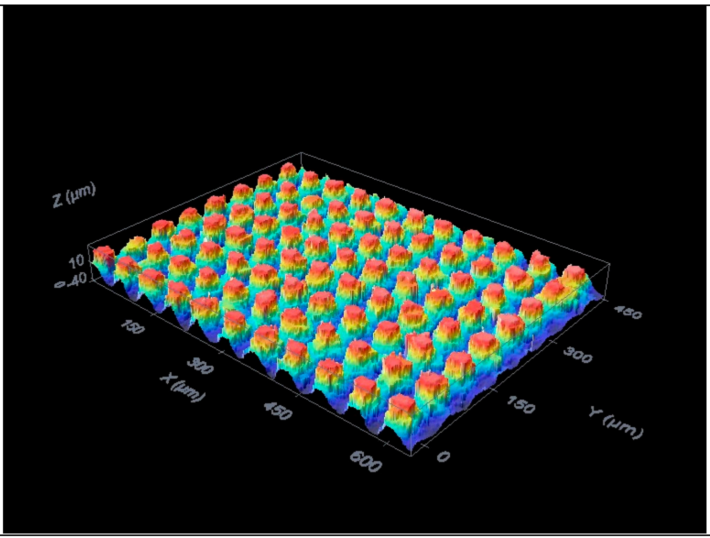
</td><td>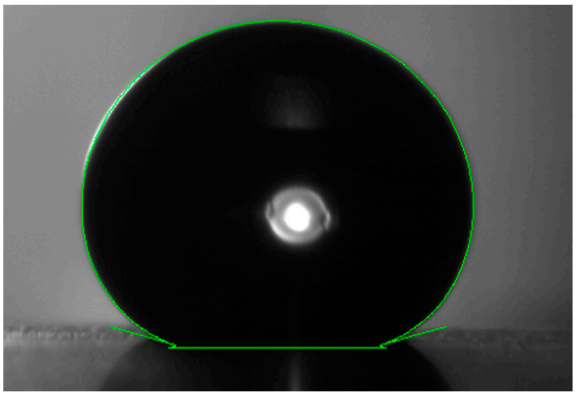</td></tr>
</table>

<table>
<tr><td colspan="2">E_{pulse} = 48.5 µJ/pulse, v_{laser} = 9 mm/s, $\langle F_{1D}\rangle$ = 107,7 J/cm^2, d_{lines} = 65 µm</td></tr>
<tr><td>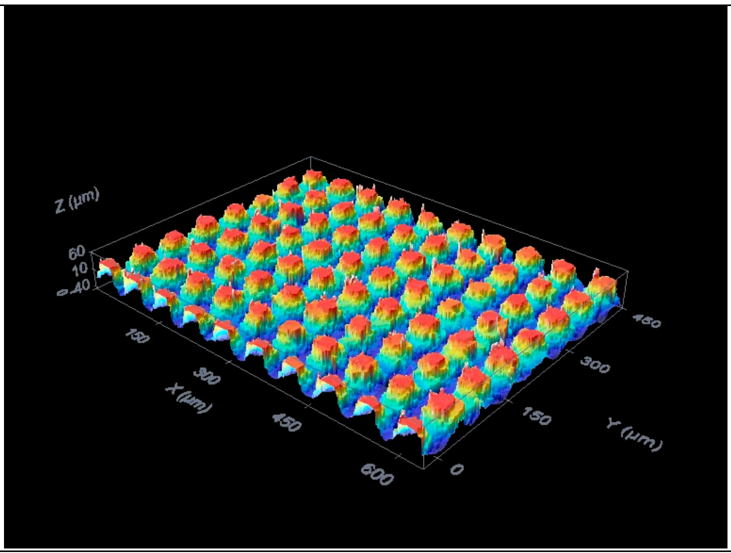
</td><td>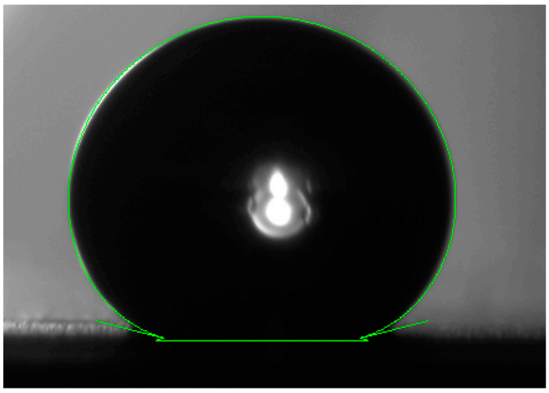</td></tr>
<tr><td colspan="2">E_{pulse} = 48.5 µJ/pulse, v_{laser} = 9 mm/s, $\langle F_{1D}\rangle$ = 107,7 J/cm^2, d_{lines} = 70 µm</td></tr>
<tr><td>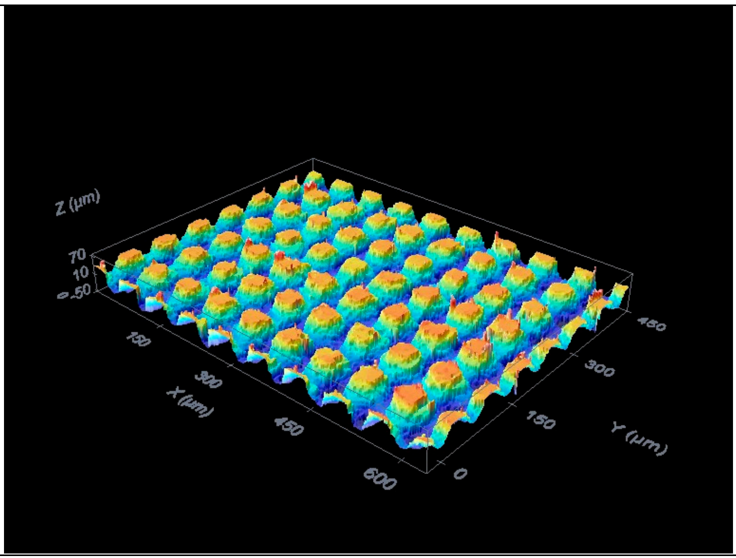
</td><td>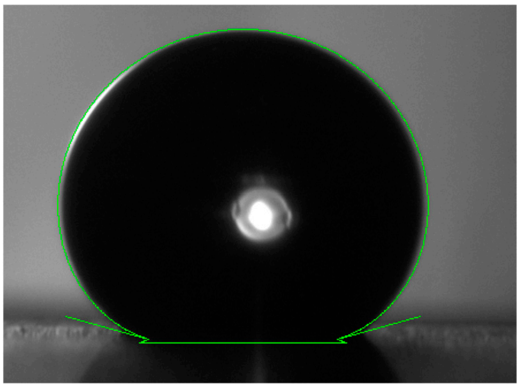</td></tr>
<tr><td colspan="2">E_{pulse} = 48.5 µJ/pulse, v_{laser} = 9 mm/s, $\langle F_{1D}\rangle$ = 107,7 J/cm^2, d_{lines} = 200 µm</td></tr>
<tr><td>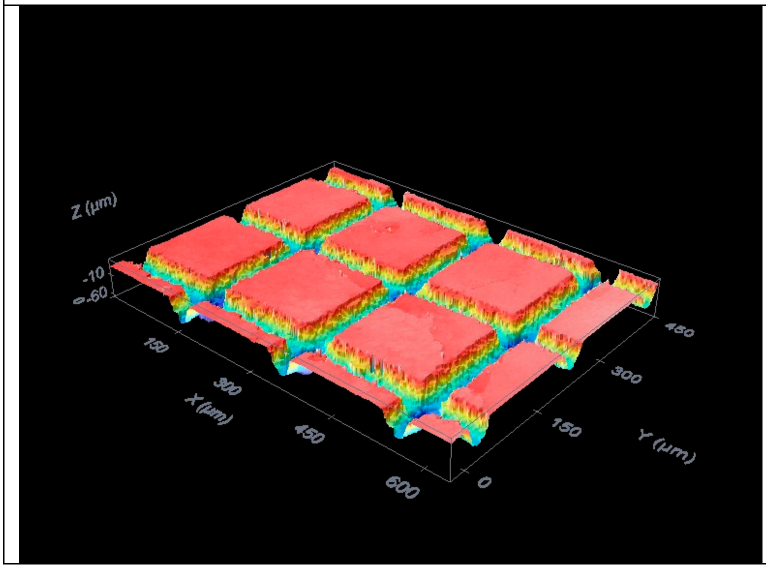
</td><td>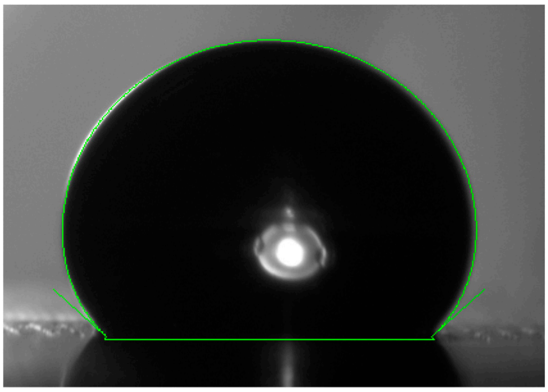</td></tr>
</table>

<table>
<tr><td colspan="2">E_{pulse} = 38,7 µJ/pulse, v_{laser} = 9 mm/s, $\langle F_{1D} \rangle$ = 85,9 J/cm^2, d_{lines} = 65 µm</td></tr>
<tr><td>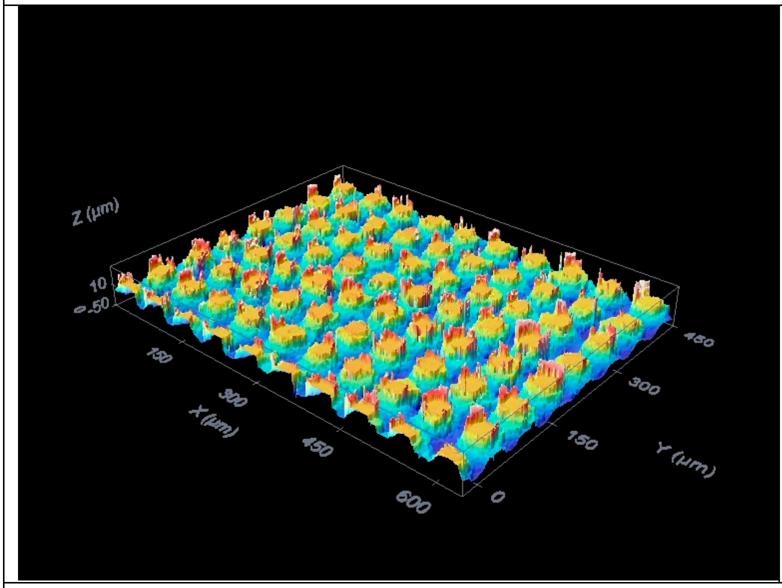
</td><td>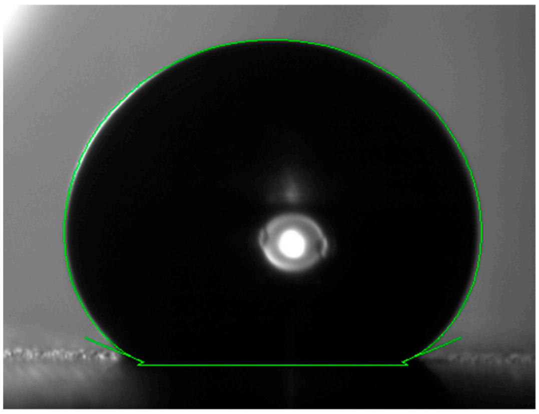</td></tr>
<tr><td colspan="2">E_{pulse} = 31 µJ/pulse, v_{laser} = 9 mm/s, $\langle F_{1D} \rangle$ = 68,9 J/cm^2, d_{lines} = 65 µm</td></tr>
<tr><td>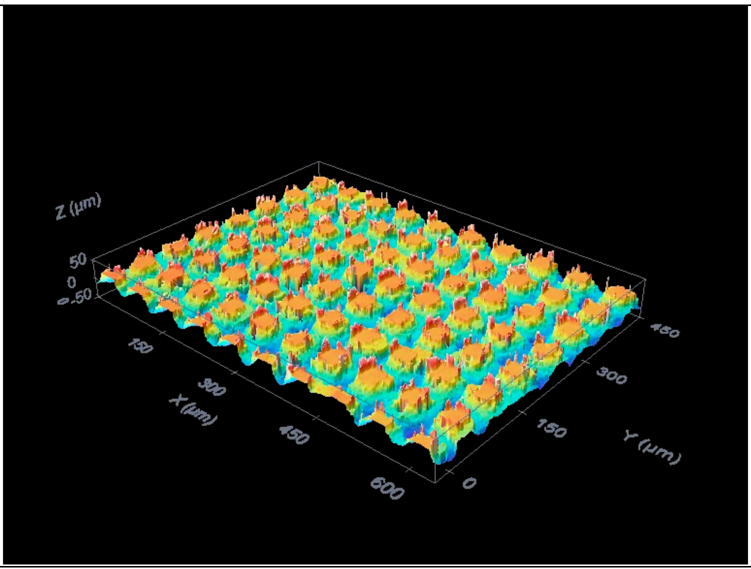
</td><td>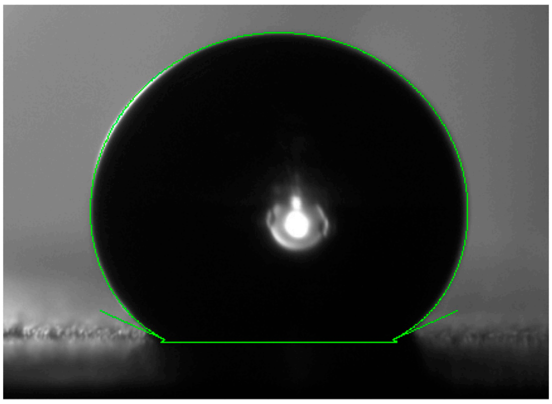</td></tr>
<tr><td colspan="2">E_{pulse} = 24,3 µJ/pulse, v_{laser} = 9 mm/s, $\langle F_{1D} \rangle$ = 54 J/cm^2, d_{lines} = 65 µm</td></tr>
<tr><td>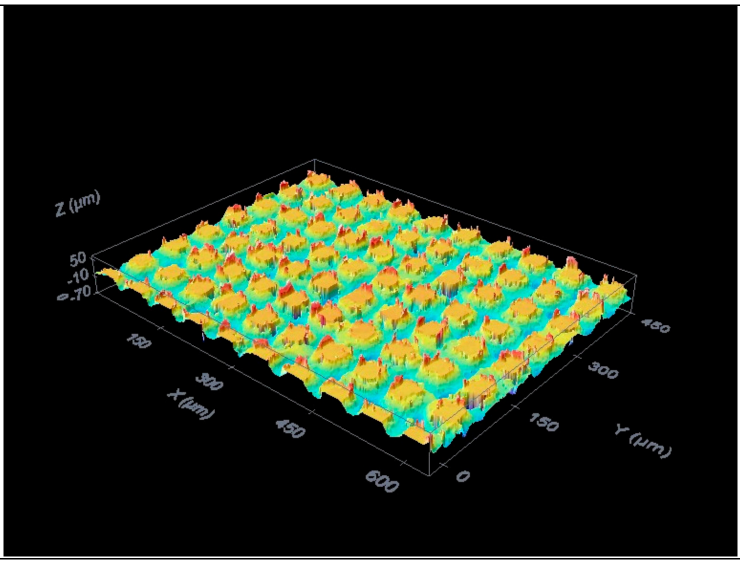
</td><td>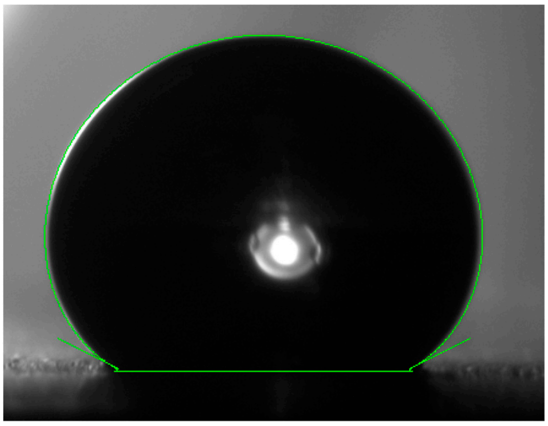</td></tr>
</table>

E_{pulse} = 18,9 µJ/pulse, v_{laser} = 9 mm/s, $\langle F_{1D} \rangle$ = 42 J/cm², d_{lines} = 65 µm	
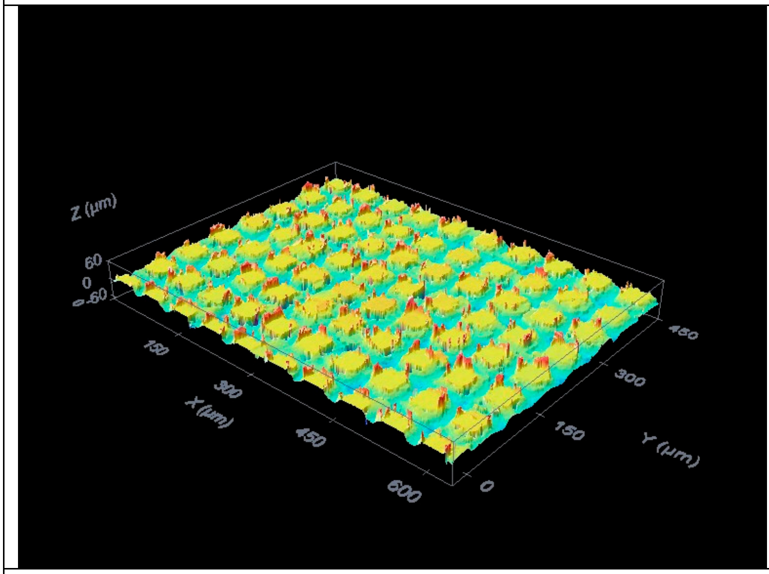	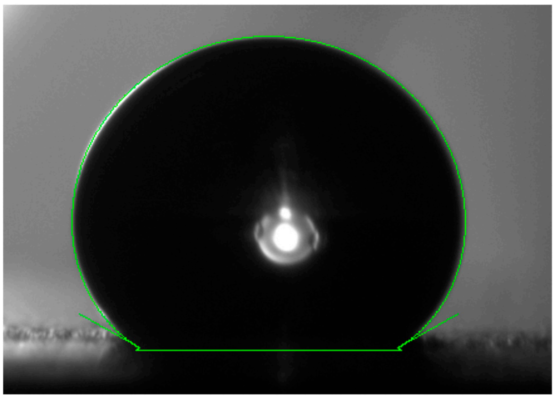
E_{pulse} = 13,6 µJ/pulse, v_{laser} = 9 mm/s, $\langle F_{1D} \rangle$ = 30,1 J/cm², d_{lines} = 65 µm	
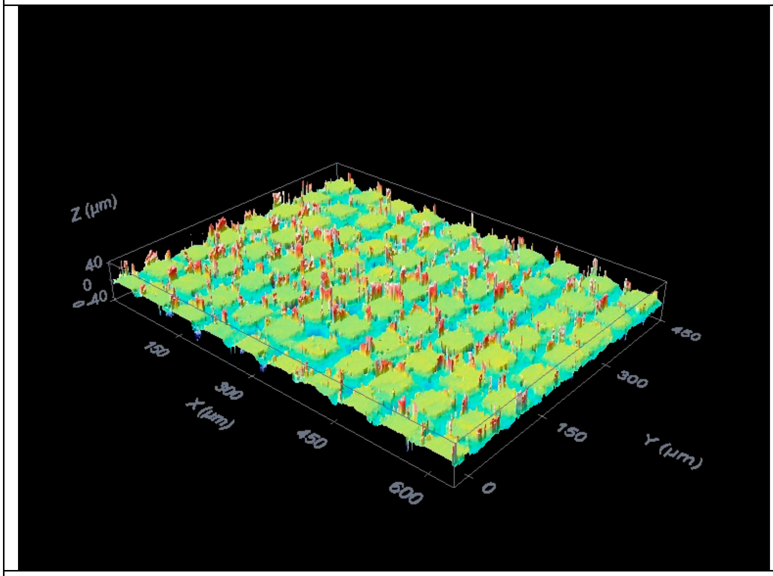	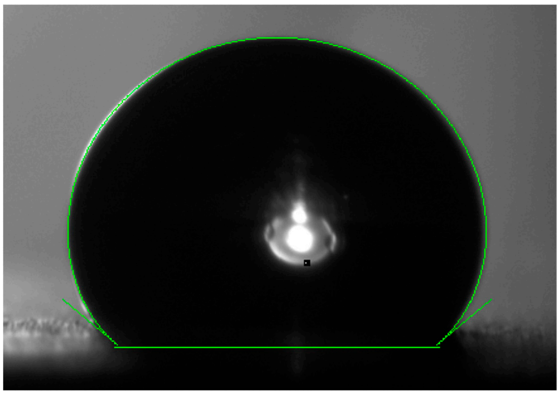
E_{pulse} = 48.5 µJ/pulse, v_{laser} = 18 mm/s, $\langle F_{1D} \rangle$ = 53,8 J/cm², d_{lines} = 65 µm	
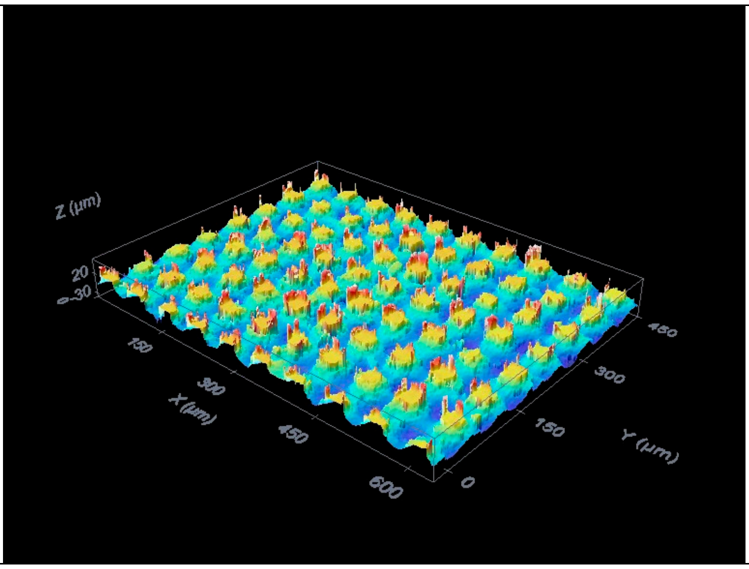	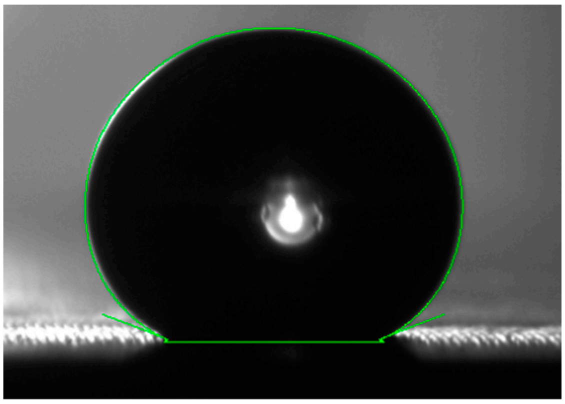

E_{pulse} = 48.5 μJ/pulse, v_{laser} = 27 mm/s, $\langle F_{1D} \rangle$ = 35,9 J/cm^2, d_{lines} = 65 μm

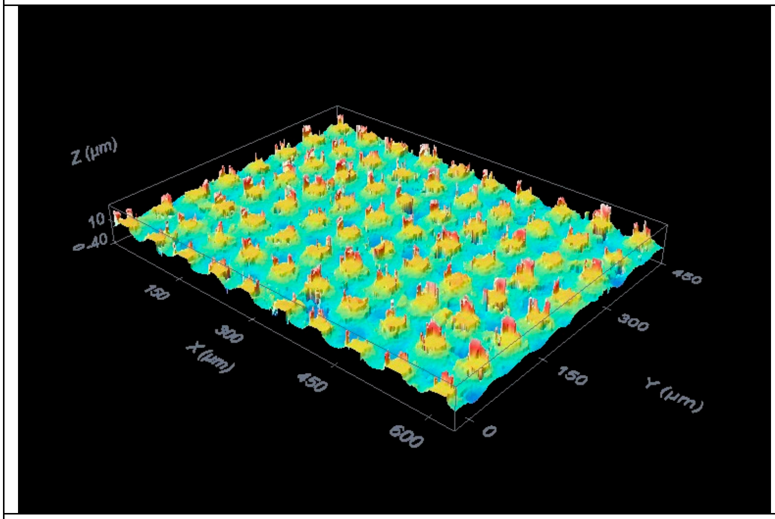

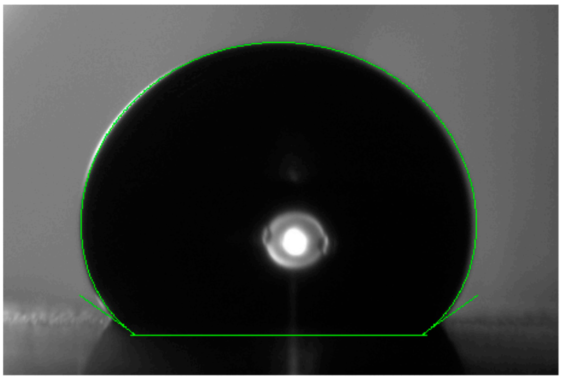

E_{pulse} = 48.5 μJ/pulse, v_{laser} = 36 mm/s, $\langle F_{1D} \rangle$ = 26,9 J/cm^2, d_{lines} = 65 μm

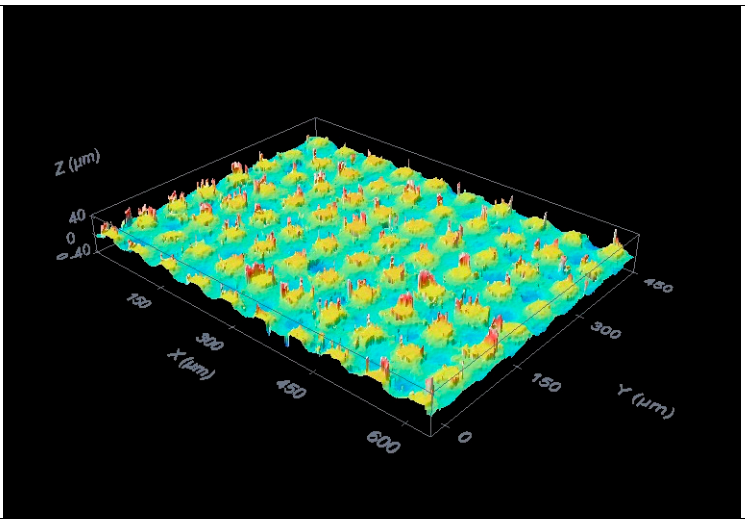

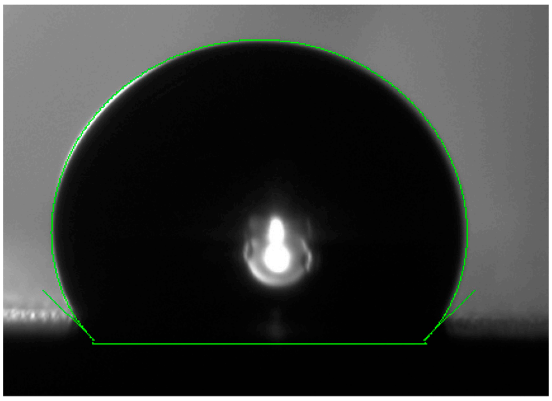

E_{pulse} = 48.5 μJ/pulse, v_{laser} = 45 mm/s, $\langle F_{1D} \rangle$ = 21,5 J/cm^2, d_{lines} = 65 μm

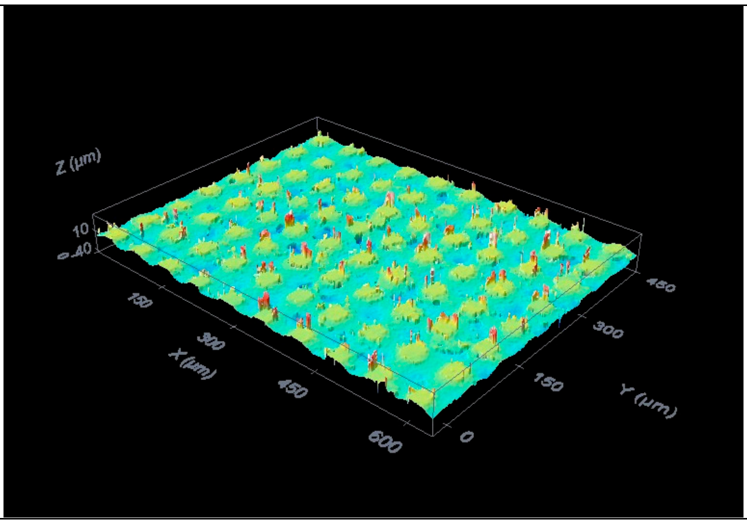

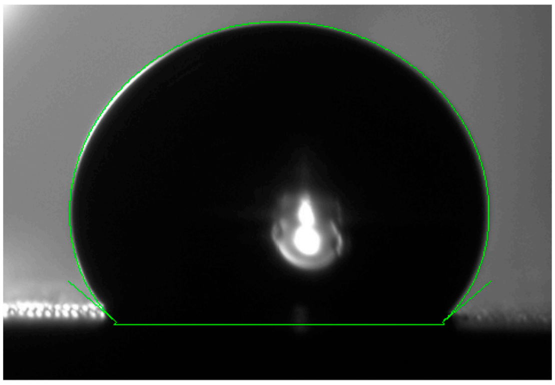

Figure S1:

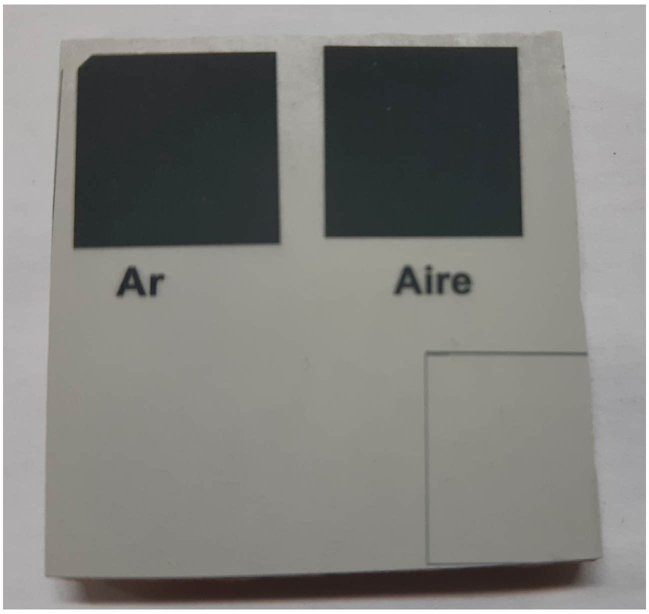

Figure S1. Photograph of the sample surface after performing the same laser treatment in air and in Ar. Experiments were carried out with E_{pulse} = 48.5 μJ/pulse, v_{laser} = 9 mm/s and and d_{lines} = 65 μm. Sample dimensions are 4 cm × 4 cm and the laser treated regions are 1.5 cm × 1.5 cm.

Figure S2:

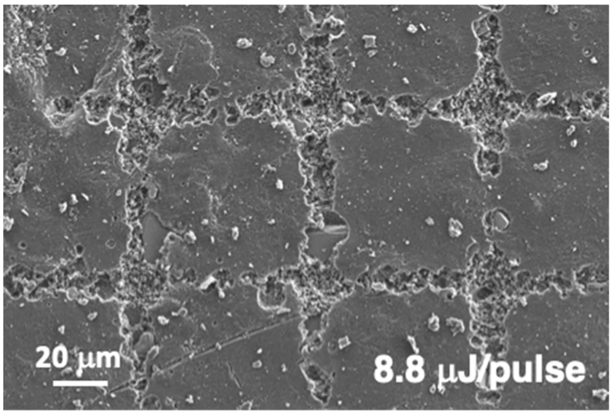

Figure S2. FESEM images of the processed sample surface with E_{pulse} = 8.8 μJ/pulse. Other laser processing parameters included v_{laser} = 9 mm/s and d_{lines} = 65 μm.
